# Malignant Degeneration of Biopsy-Proven Hamartoma to Chondrosarcoma

**DOI:** 10.7759/cureus.12150

**Published:** 2020-12-18

**Authors:** Rachel Schenkel, Charles Altfillisch, Janice Chung, Ankit Verma, Marcus Balters

**Affiliations:** 1 Surgery, Creighton University School of Medicine, Omaha, USA

**Keywords:** hamartoma, chondrosarcoma, transformation, degeneration, dedifferentiation, pulmonary

## Abstract

Pulmonary hamartomas are benign lesions that are often managed conservatively in the absence of respiratory symptoms. Increasing reports of malignant transformation question if a more aggressive treatment or surveillance practice for these lesions is warranted in adult patients. Herein, we describe a case of a 67-year-old man with a long history of pulmonary hamartoma that demonstrated malignant degeneration into spindle cell malignancy with chondromatous differentiation. This case illustrates the aggressive nature of sarcomatous disease arising from hamartomas and, with a handful of other cases in the literature, points to the question of whether pulmonary hamartomas arising in late adulthood should follow a more intensive treatment or surveillance algorithm given increased concern for malignant potential.

## Introduction

Hamartomas are benign tumors that maintain the characteristic tissue of the skin or soft tissue site at which they are located, albeit in a disordered fashion [1]. There are several categories of hamartomas [1], including the pulmonary hamartoma [2-5]. This subtype is predominately seen in adult men, often in the fourth to seventh decades of life [3,4], who usually have smoking exposure [3]. Diagnosis is frequently due to incidental findings on chest imaging, although some have been preceded by respiratory symptoms such as coughing and hemoptysis [3,6]. While these lesions tend to start small and grow slowly, they can become large and symptomatic or concerning for malignancy [2], in which case surgical excision may be warranted [7] - otherwise, in the absence of such, conservative management with serial imaging is recommended [8]. Specifically, a study has suggested that positron emission tomography-computed tomography (PET-CT) was better able to determine if a lesion was benign in comparison to CT, although those deemed suspicious for malignancy based on PET-CT were found to be histologically benign [9]. While these tumors are typically histologically devoid of neoplastic characteristics [5,10,11], increasing literature is describing cases of lesions evolving to malignant chondrosarcoma [5,12-14] and concurrence with primary lung carcinomas [11,15]. Chondrosarcomas are most commonly low-grade primary neoplasms of the bone that rarely metastasize, although pulmonary metastatic disease can occur and portends a poor prognosis [16,17]. Treatment recommendations regarding indications and timing of resection are controversial [18,19]. Herein, we describe a case of an elderly man who presented with biopsy-proven hamartoma that evolved to chondrosarcoma. 

## Case presentation

**Figure 1:**
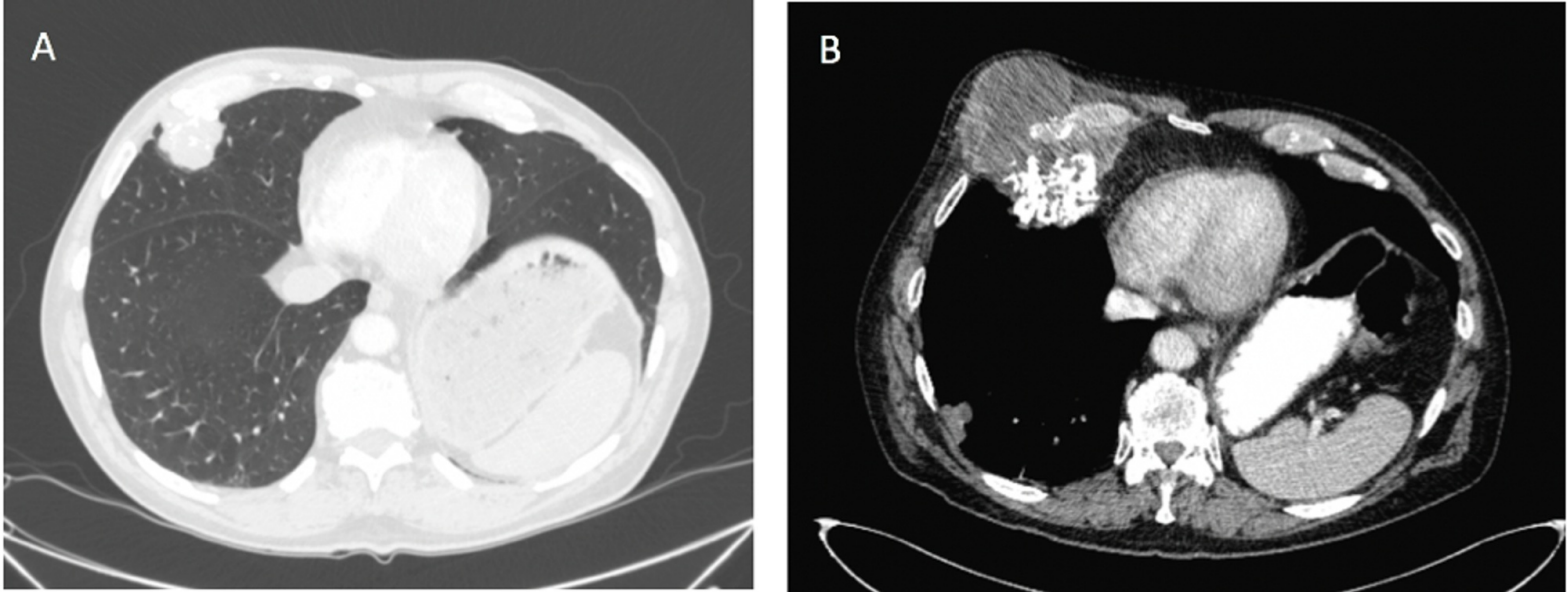
The patient’s right middle lobe mass demonstrated on CT in 2012 (A) and in June 2018 (B).

A 67-year-old gentleman who presented to thoracic surgery clinic in 2017 for evaluation of an enlarging, partially calcified right middle lobe mass, found incidentally on CT in 2012. In 2012, his mass measured 3.0x3.3 cm and was evaluated with PET-CT, which read this mass as mildly hypermetabolic and likely a hamartoma, though due to its position interfacing with an adjacent rib, a rib chondrosarcoma could not be ruled out (Table 1; Figure 1A). 

**Table 1:** Patient’s lesion size throughout the years.

Date	Size/Additional Information
March 2012	3.3x3.6 cm
May 2013	3.3x4.0 cm
May 2014	4.9x3.9 cm
May 2015	5.1x4.7 cm
July 2016	5.6x5.6 cm
June 2017	5.9x5.8x6 cm - radiologist read recommended surgical consultation at this time
July 2017	First Thoracic Surgery clinic visit, patient and team agreed to observation
February 2018	6.6x6.5 cm - impression read "unchanged large partially calcified upper lobe mass, stable since June 2017, but enlarged since 2012"
June 2018	9.6x7.9 cm - small pulmonary nodules seen at this time
July 2018	Rebiopsied showing sarcoma with metastatic disease
August 2018	PET showed increase in size of small pulmonary nodules seen in June (mets)
November 2018	9.7x7.7 cm
January 2019	7x5.3 cm
March 2019	8.4x7.1 cm

**Figure 2:**
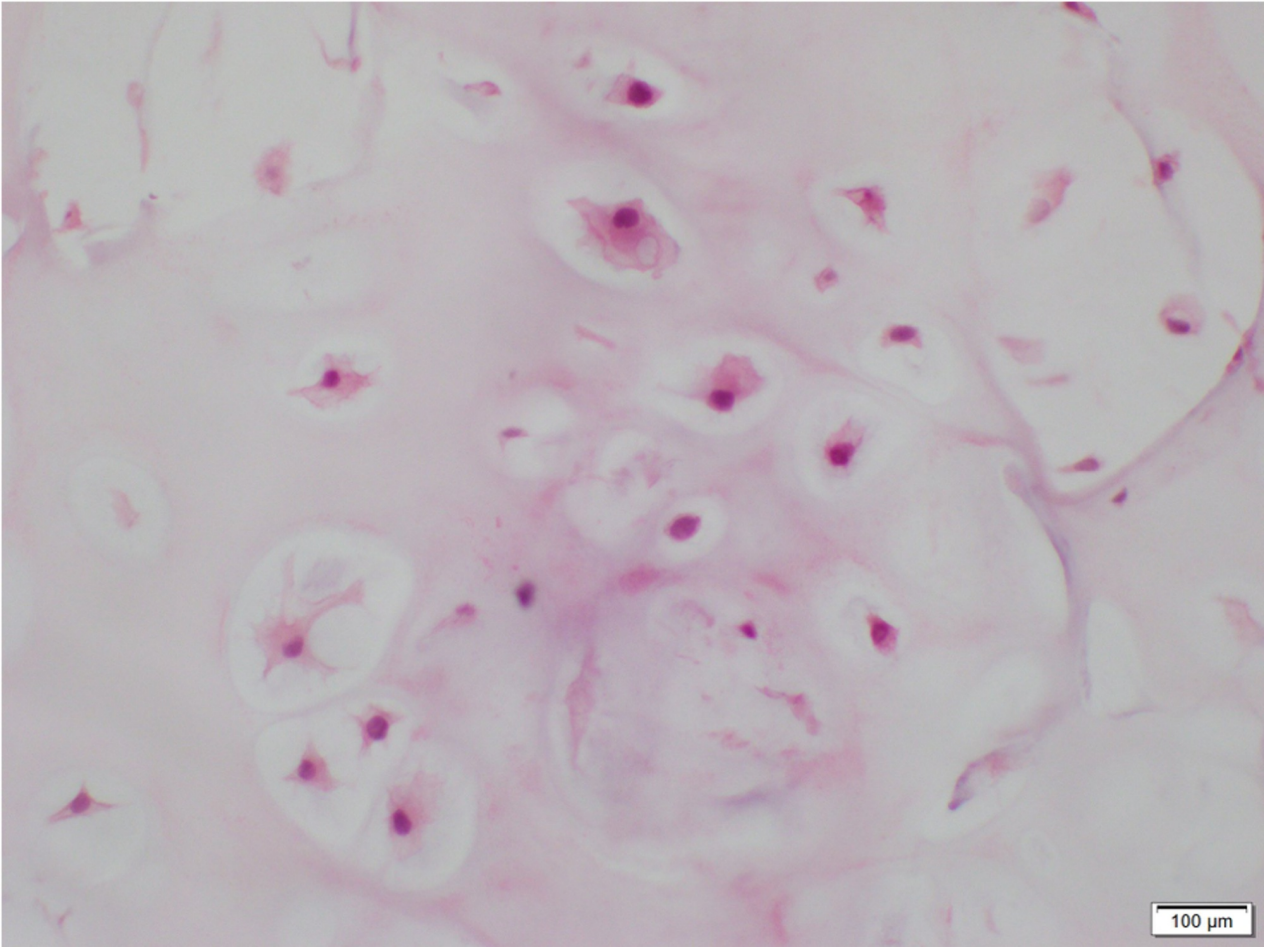
Histology of the patient’s right middle lobe mass in 2012 showing a mixture of benign cartilage with scant fragments of bone and adipose tissue. Calcifications are noted. There is no evidence of cytologic atypia or malignancy identified.

**Figure 3:**
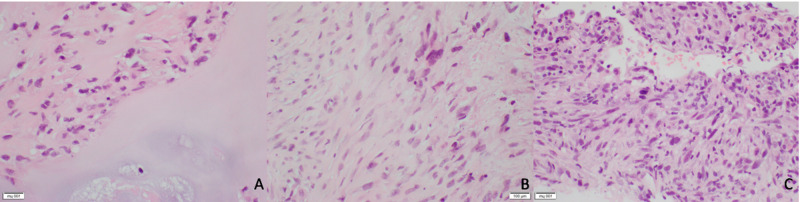
Histology of the patient’s lesions in 2018: right chest wall/lung mass (A) showing well-differentiated cartilage with focal atypical spindle cells, and right lung nodule (B,C) showing malignant spindle cell neoplasm.

Biopsy was then performed which confirmed diagnosis of hamartoma with no evidence of cytologic atypia or malignancy identified (Figure 2). Steady growth prompted patient’s referral to the surgery clinic in 2017, and upon shared decision-making, the patient and team agreed upon observation. He was surveilled with yearly CTs following this, with regular follow up appointments and stable symptoms. His mass demonstrated slow, steady enlargement until June 2018 when it grew rapidly to 9.6x7.9 cm with a new large component extending into the chest wall and subcutaneous tissue and several new lung nodules consistent with metastatic disease (Figure 1B). He underwent re-biopsy of both the chest wall mass and lung lesion at this time and these showed spindle cell malignancy with chondromatous differentiation (Figure 3). PET-CT demonstrated multiple bilateral increased hypermetabolic pulmonary nodules. Due to the metastatic nature of his disease, he was no longer a surgical candidate and began chemotherapy, receiving four cycles of doxorubicin and one dose of olaratumab to which he had an anaphylactic reaction. Chemotherapy did not have a significant effect on his disease burden, and he was offered palliative external beam radiation. He passed away from his disease shortly after this.

 

## Discussion

In our case, the patient had previously had biopsy confirmed hamartoma which then rapidly degenerated to chondrosarcoma despite surveillance. It is difficult to pinpoint any predicting factor that may have helped prevent mortality in our patient. We have reviewed similar cases that have been presented in the literature. A 74-year-old woman was found to have a biopsy-proven left-sided pulmonary chondroid hamartoma that transformed to intermediate-grade chondrosarcoma over the course of seven years after radiation therapy for breast cancer [12]. Similarly, in Japan, a 73-year-old male presented with a left-sided hamartomatous lung nodule that was excised due to large size. Initially, the biopsy was not diagnostic, but on excision the histologic changes were suggestive of low-grade chondrosarcoma evolving into fibrosarcoma [13]. An Italian 62-year-old woman possessed a right-sided pulmonary hamartoma that was resected via lobectomy due to effusion 37 years after the discovery of the lesion, at which point no neoplastic changes were found on histology. However, at autopsy three months later, histology was suggestive of a primary pleomorphic sarcoma [14]. We suggest that cases of pulmonary hamartomas arising in late adulthood are more concerning for future malignant degeneration.

Enlarging nodules, even with the diagnosis of hamartoma, should be excised. When patients are not surgical candidates or are averse to excision, they should have a different algorithm for treatment involving repeat biopsy and serial CTs. If enlargement continues, stronger recommendations for excision should be given. We suggest imaging every six months for two years, then yearly through year five, then every two years until year ten. If no changes occur during this surveillance, imaging can be discontinued at that time, although there are no randomized studies to validate this. If changes are found, again, we recommend rebiopsy or excision given the risks we are describing. If the patient becomes symptomatic from their hamartoma after their period of surveillance, we also recommend rebiopsy or excision at that time. If clinical suspicion outweighs benign biopsy results or negative PET scans, rebiopsy or excision should be considered. Understandably, reactive decision-making can prove to be difficult, but patient goals and quality of life need to be taken into consideration as well. Needless removal of a large lesion can significantly deteriorate patient quality of life and has intraoperative risks as well. 

We hope to draw the attention of the thoracic community to the malignant potential of these classically benign pulmonary lesions, and to recommend a low threshold for excision. Early referral of patients with these lesions to thoracic surgery is of increased importance in light of the increasing case reports of sarcomatous transformation. Guo, et al. recommended wedge resection in uncomplicated cases, so as to reserve lobectomy or pneumonectomy for cases in which the tumors were deep and invading the hilum, involved tissue no longer provided respiratory function, or severe disease burden. They also noted the importance of procuring frozen sections during operative procedures in order to better grade the tumor [3].

## Conclusions

Pulmonary hamartomas are considered to have a good prognosis due to their slow growth and extremely low metastatic potential. However, rare cases of rapid expansion are reported in the literature, which should prompt a thorough malignancy workup as hamartomas have been associated with an increased rate of concurrent primary lung carcinoma. Despite this, malignant degeneration of pulmonary hamartomas is exceedingly rare and treatment recommendations regarding indications and timing of resection are controversial. We suggest that enlarging pulmonary hamartomas arising in adulthood should be resected in fit patients or aggressively surveilled and re-biopsied at regular intervals in patients who are less fit surgical candidates.
